# Supplementary material for: Evolutionary paths toward multi-level convergence of lactic acid bacteria in fructose-rich environments
Source: Commun Biol. 2024 Jul 24;7:902. doi: 10.1038/s42003-024-06580-0 (PMC11269746; doi:10.1038/s42003-024-06580-0)
Supplement: Supplementary file 2 — Supplementary Information [file 42003_2024_6580_MOESM2_ESM.pdf]

SUPPLEMENTARY MATERIALS

Title: Evolutionary paths toward multi-level convergence of lactic acid bacteria in fructose-rich environments

Running Title: Evolutionary paths toward multi-level convergence

Authors: Naoki Konno, Shintaro Maeno, Yasuhiro Tanizawa, Masanori Arita, Akihito Endo, and Wataru Iwasaki

TABLE OF CONTENTS

1. SUPPLEMENTARY FIGURES (Included in this PDF)

Supplementary Figures 1-9

2. SUPPLEMENTARY DATA (Not included in this PDF; Provided as a FASTA (.faa) file or an XLSX file.)

Supplementary Data 1. Amino acid sequences of 399 *adhE*-like genes identified in this paper and of one reference *adhE* gene of *Escherichia coli*

Supplementary Data 2. Reference genome accession, AdhE possession, genome statistics, and fermentation type of Lactobacillaceae species analyzed in this study

Supplementary Data 3. The list of 137 orthologs commonly and independently lost in two FLAB lineages

Supplementary Data 4. The source data behind the figures in this paper

SUPPLEMENTARY FIGURES

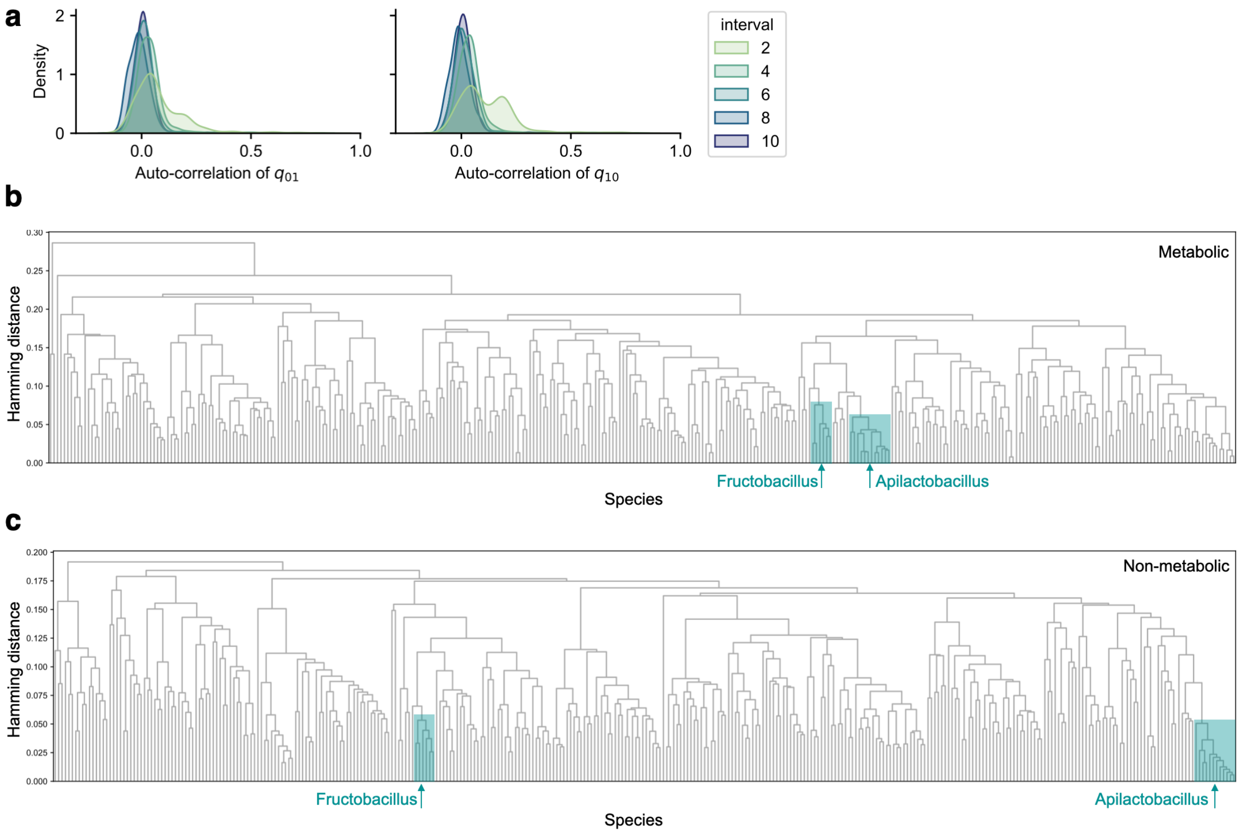

Supplementary Figure 1. **Validity confirmation of the MCMC sampling interval and the hierarchical clustering of gene contents for Lactobacillaceae species. a**, Distribution of autocorrelation coefficients of gene gain/loss rate parameters (q_{01}, q_{10}) estimated by MCMC method with continuous sampling for each sampling interval. **b**, **c**, Dendrograms of metabolic (**b**) or non-metabolic (**c**) gene sets possessed by 344 Lactobacillaceae species with high-quality representative genomes. Gene presence/absence of an OG was represented as one and zero to treat the gene content of a species as a vector. A hierarchical clustering was then conducted by the average linkage method for hamming distances among gene contents.

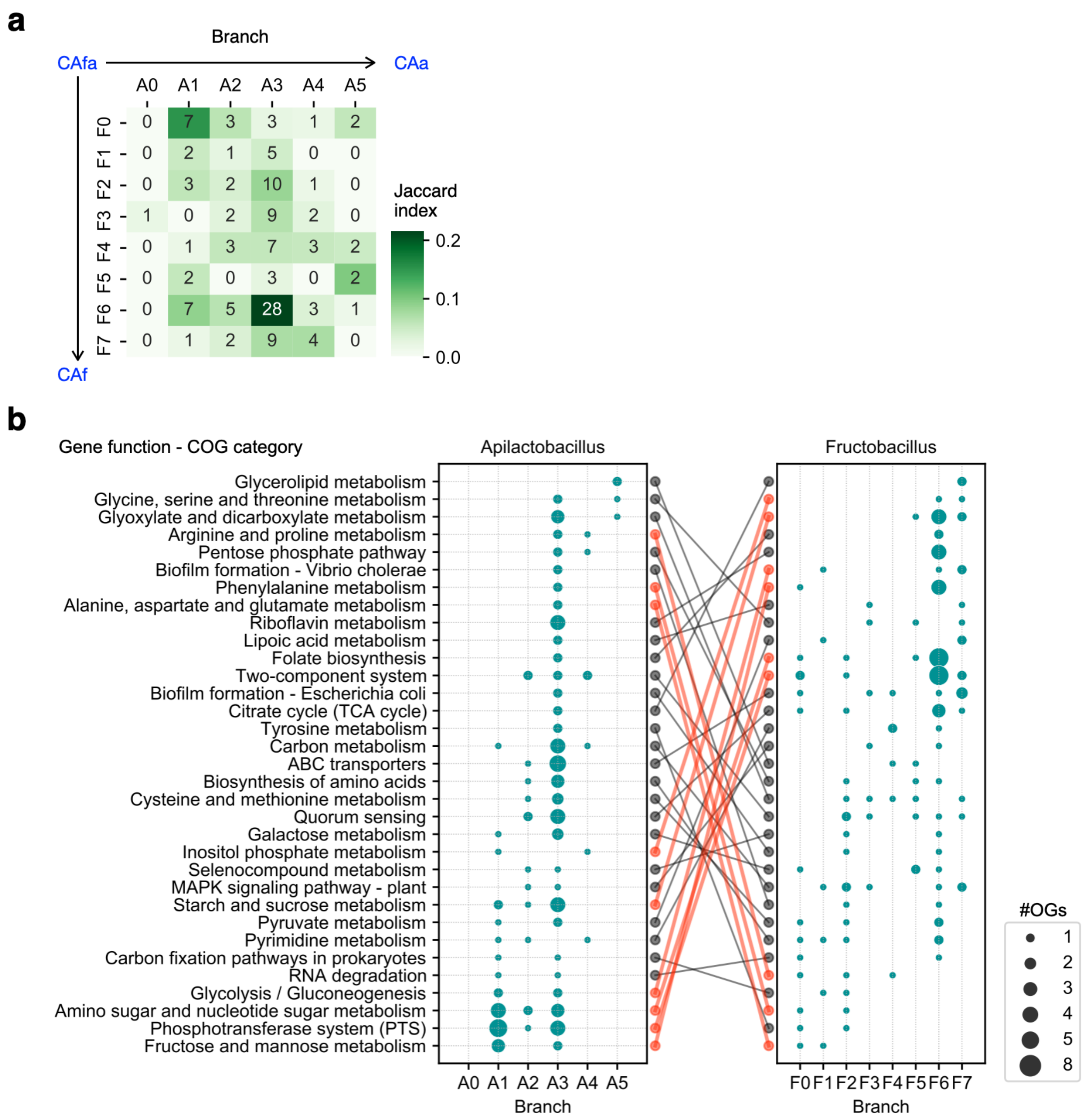

Supplementary Figure 2. Robustness confirmation of results on the significant gene loss order similarity and the loss orders of gene functions. a, Overlaps between OGs that were lost at each branch in paths toward the two FLAB lineages. The heatmap's numbers and colors indicate the numbers of commonly lost OGs and Jaccard indices between lost OG sets, respectively. **b**, KEGG-Pathway-wise counts of OGs that were lost at each branch. Only OGs commonly lost in the two lineages were included in the

counts. The KEGG Pathways were sorted by the average timing of gene losses in each lineage. Red and grey edges connect the same pathway, showing markedly different or similar relative timing of gene losses.

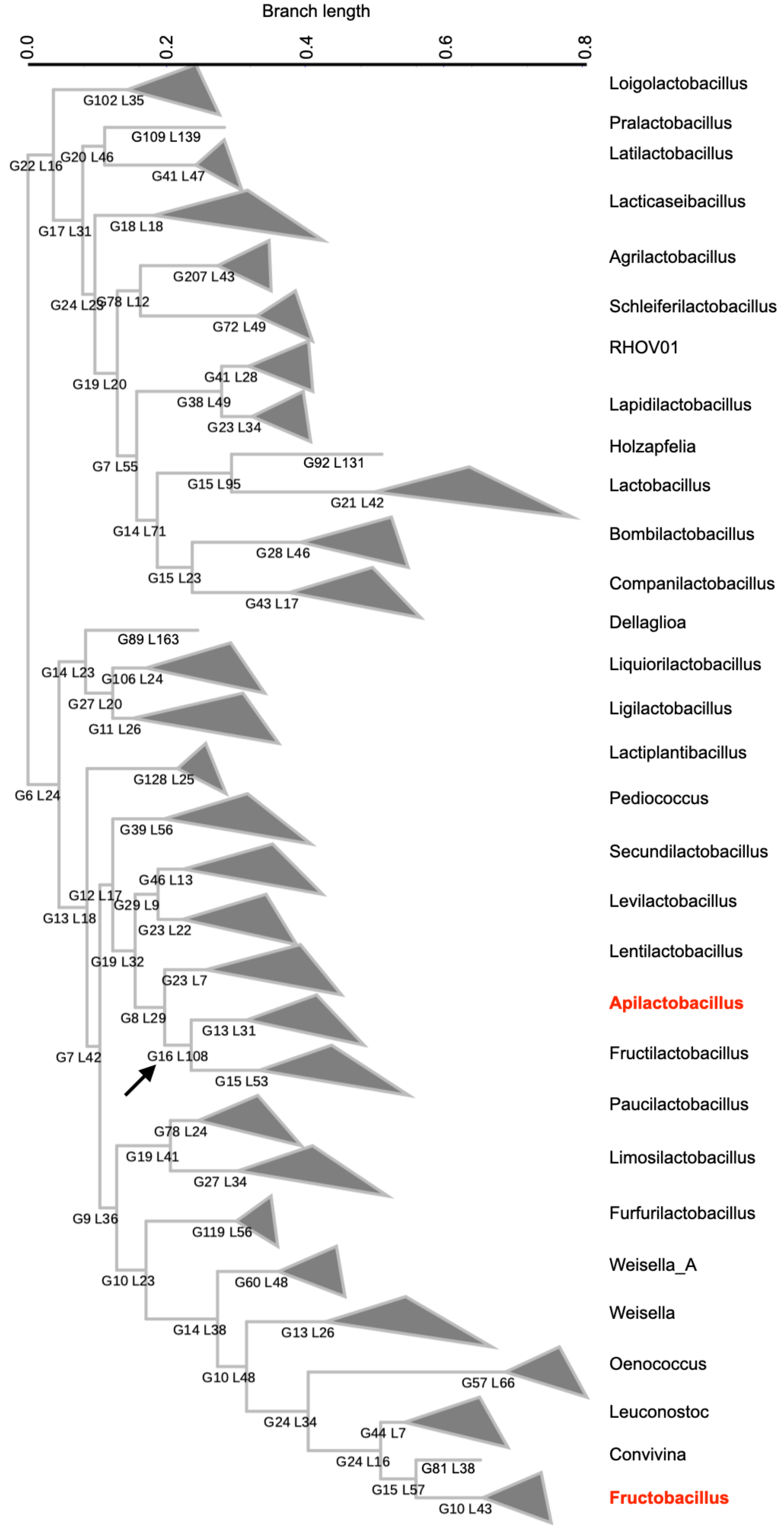
Branch length
0.0
0.2
0.4
0.6
0.8
G102 L35
G22 L16
G20 L46
G109 L139
G41 L47
G17 L31
G18 L18
G207 L43
G24 L25
G78 L12
G72 L49
G19 L20
G41 L28
G38 L49
G23 L34
G7 L55
G92 L131
G15 L95
G21 L42
G14 L71
G28 L46
G15 L23
G43 L17
G89 L163
G14 L23
G106 L24
G27 L20
G11 L26
G6 L24
G128 L25
G39 L56
G46 L13
G12 L17
G29 L9
G13 L18
G23 L22
G19 L32
G23 L7
G8 L29
G13 L31
G7 L42
G16 L108
G15 L53
G78 L24
G19 L41
G27 L34
G9 L36
G119 L56
G10 L23
G60 L48
G14 L38
G13 L26
G10 L48
G57 L66
G24 L34
G44 L7
G24 L16
G81 L38
G15 L57
G10 L43
Loigolactobacillus
Pralactobacillus
Latilactobacillus
Lacticaseibacillus
Agrilactobacillus
Schleiferilactobacillus
RHOV01
Lapidilactobacillus
Holzapfelia
Lactobacillus
Bombilactobacillus
Companilactobacillus
Dellaglioa
Liquiorilactobacillus
Ligilactobacillus
Lactiplantibacillus
Pediococcus
Secundilactobacillus
Levilactobacillus
Lentilactobacillus
Apilactobacillus
Fructilactobacillus
Paucilactobacillus
Limosilactobacillus
Furfurilactobacillus
Weisella_A
Weisella
Oenococcus
Leuconostoc
Convivina
Fructobacillus

Supplementary Figure 3. The number of orthologs gained/lost at every branch of the phylogeny. The GTDB-based genus-level phylogeny of Lactobacillaceae is shown with the number of gained and lost orthologs at each branch. "G*x* L*y*" written below a branch represents *x* and *y* orthologs were gained and lost at the branch, respectively. The names of FLAB genera are colored red. The arrow indicates the branch where massive gene loss occurred as mentioned in the Result section.

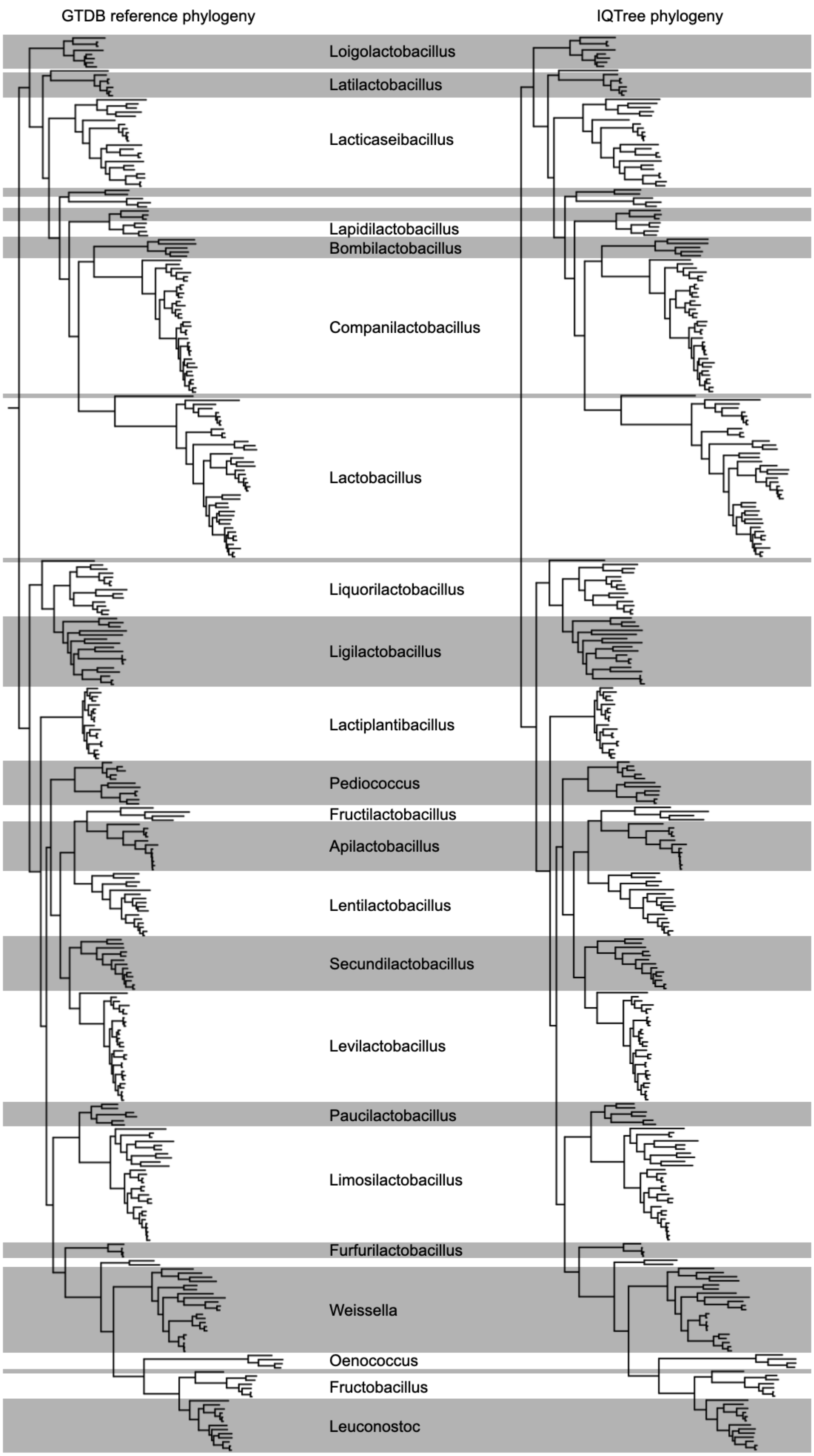

GTDB reference phylogeny
IQTree phylogeny
Loigolactobacillus
Latilactobacillus
Lacticaseibacillus
Lapidilactobacillus
Bombilactobacillus
Companilactobacillus
Lactobacillus
Liquorilactobacillus
Ligilactobacillus
Lactiplantibacillus
Pediococcus
Fructilactobacillus
Apilactobacillus
Lentilactobacillus
Secundilactobacillus
Levilactobacillus
Paucilactobacillus
Limosilactobacillus
Furfurilactobacillus
Weissella
Oenococcus
Fructobacillus
Leuconostoc

Supplementary Figure 4. Comparison of an extracted GTDB reference phylogeny and a re-inferred phylogeny. The phylogeny of 344 Lactobacillaceae species extracted from the GTDB reference phylogeny of whole bacteria (left) and the phylogeny re-inferred from the multiple sequence alignment of the same set of species using IQTree-2.0.3 (-m MFP -bb 1000 -nt 20) (right). Names of genera corresponding to clades with four or more species in the two trees are written in the middle. The normalized Robinson-Foulds distance between the two trees was 0.032.

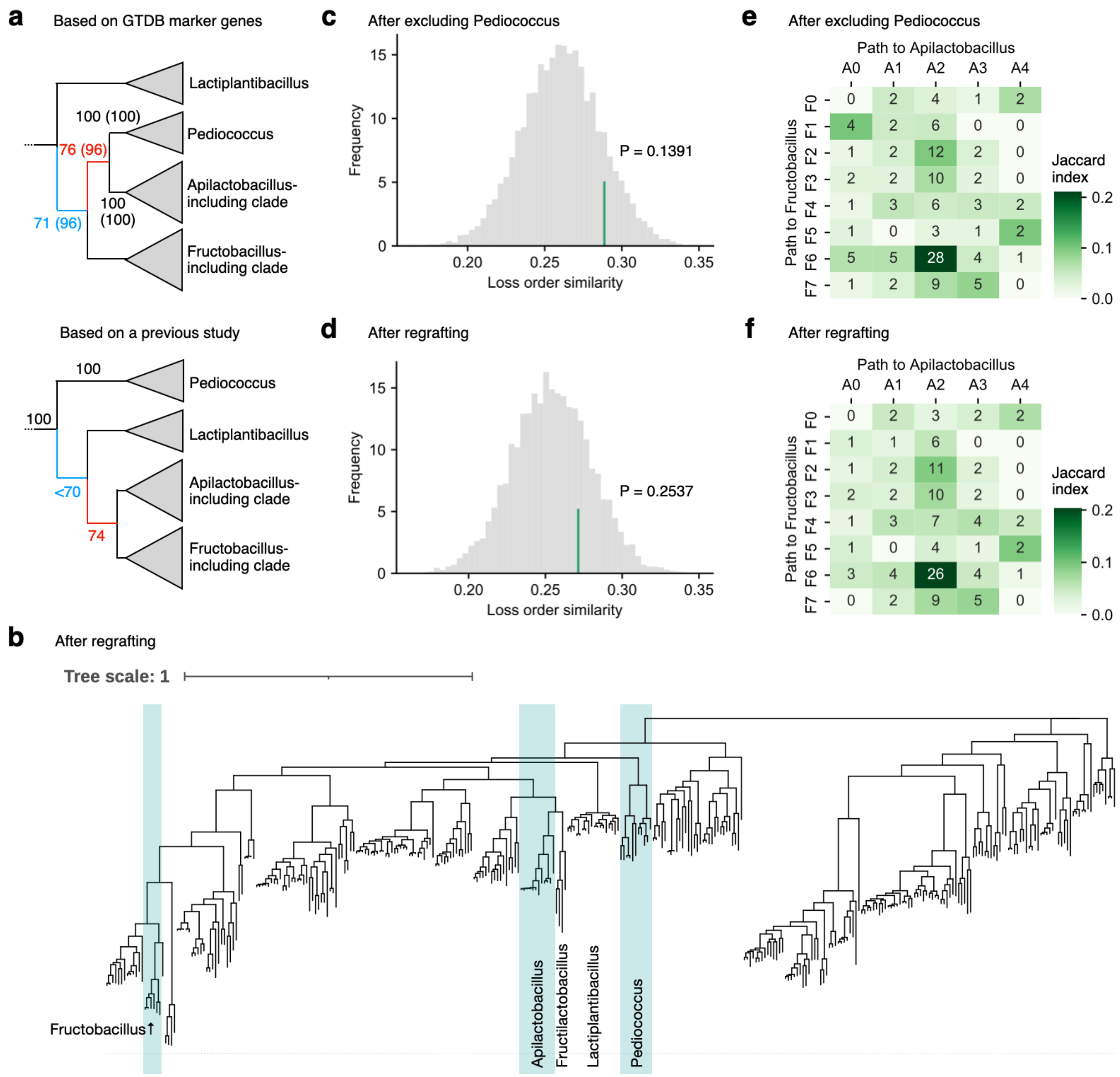

Supplementary Figure 5. Robustness test of the gene loss order similarity of FLAB against the phylogenetic position of Pediococcus. a, Comparison of Pediococcus position between the re-inferred tree from marker gene sequences provided in GTDB and a previously reported phylogeny [1]. The re-inferred tree was computed by IQTree-2.0.3 with standard bootstrapping (-m MFP -b 200 -nt 20) and ultrafast bootstrap values (-m MFP -bb 1000 -nt 20). Standard bootstrap values are written on the corresponding branches and ultrafast bootstrap values are written in the parentheses. Bootstrap values of red and blue branches are mentioned in the Results section. **b**, A tree modified from the GTDB reference phylogeny in which the

Pediococcus position was regrafted to adjust to the previously reported phylogeny [1]. **c, d,** The statistical significance of gene loss order similarity after eliminating Pediococcus clade (**c**) or regrafting the Pediococcus clade as shown in **b** (**d**). The green vertical line segment represents the observed score of gene loss order similarity calculated for all the OGs commonly and independently lost by FLAB, while the grey histogram illustrates the null distribution of the score. P-values are indicated in each figure. **e, f,** Overlaps between OGs that were lost at each branch in paths toward the two FLAB lineages after eliminating Pediococcus clade (**e**) or regrafting the Pediococcus clade as shown in **b** (**f**). The heatmap's numbers and colors indicate the numbers of commonly lost OGs and Jaccard indices between lost OG sets, respectively.

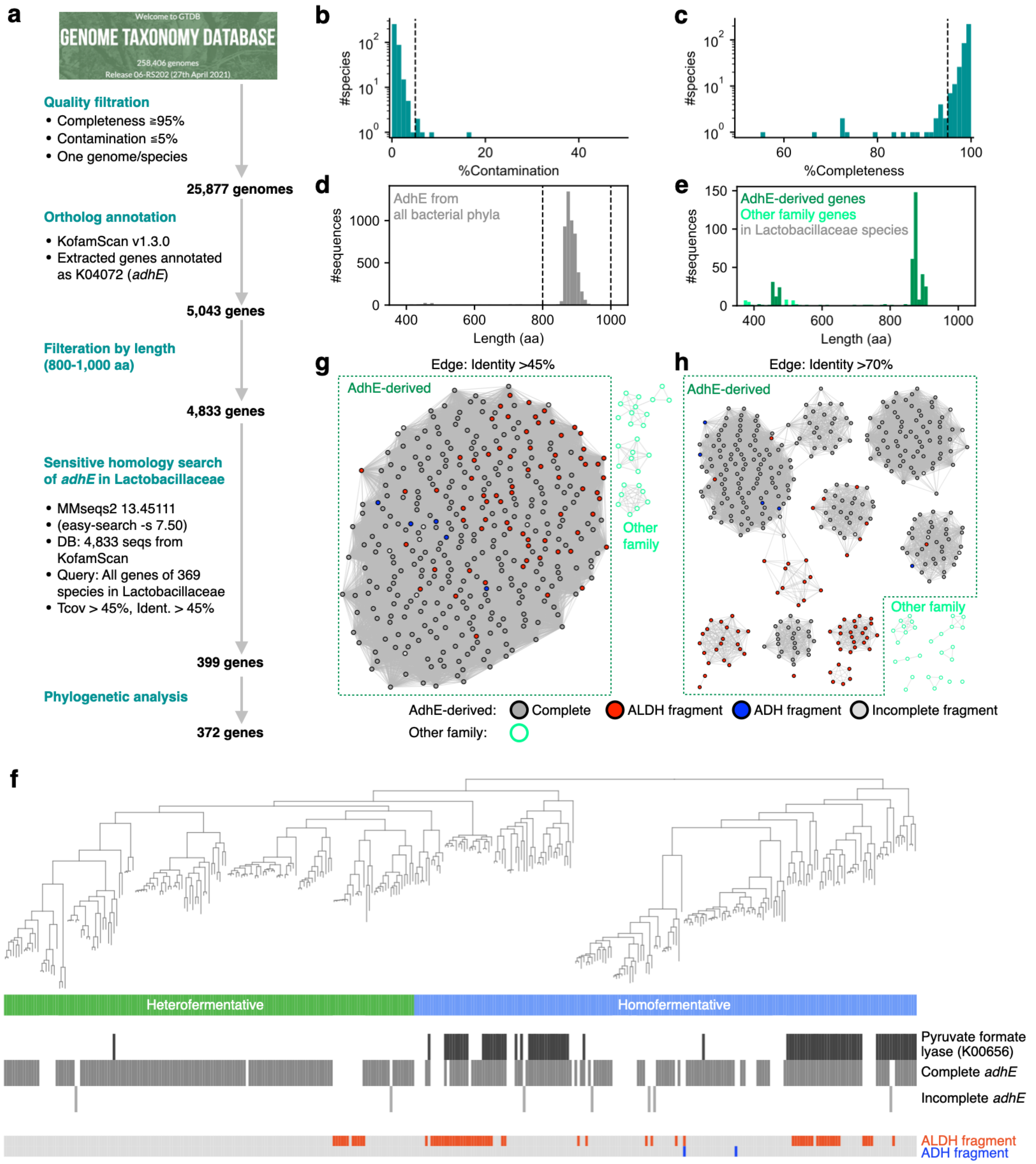

Supplementary Figure 6. Detection of complete and partial AdhE sequences. **a**, The overview of AdhE sequence detection method. **b, c,** The distribution of contamination (**b**) and completeness (**c**) of the representative genomes for 369 Lactobacillaceae species. **d**, The distribution of amino acid sequence lengths for the 5,043 AdhE-like genes detected by KofamScan against genomes of the 25,877 species in

various phyla. Six sequences longer than 1,050 aa were not indicated here. **e**, The distribution of amino acid sequence lengths for the detected 399 AdhE-like genes of Lactobacillaceae species. Green and yellow-green bars indicate AdhE-derived genes and genes in other families inferred from the phylogenetic analysis. **f,** Phylogenetic distribution of complete/fragmental AdhE and pyruvate formate lyase. The GTDB-based Lactobacillaceae phylogeny (top), the distribution of homo/heterofermentative species (green/blue) (middle), and the presence/absence profile of pyruvate formate lyases (black), complete/incomplete AdhEs (thick/thin grey), and ALDH/ADH fragments (red/blue) (bottom). The copy number is not shown in this figure. **g**, **h**, The sequence similarity networks of 399 AdhE-like genes. The edges indicate pairs of genes showing >45% (**g**) or >70% (**h**) sequence identity.

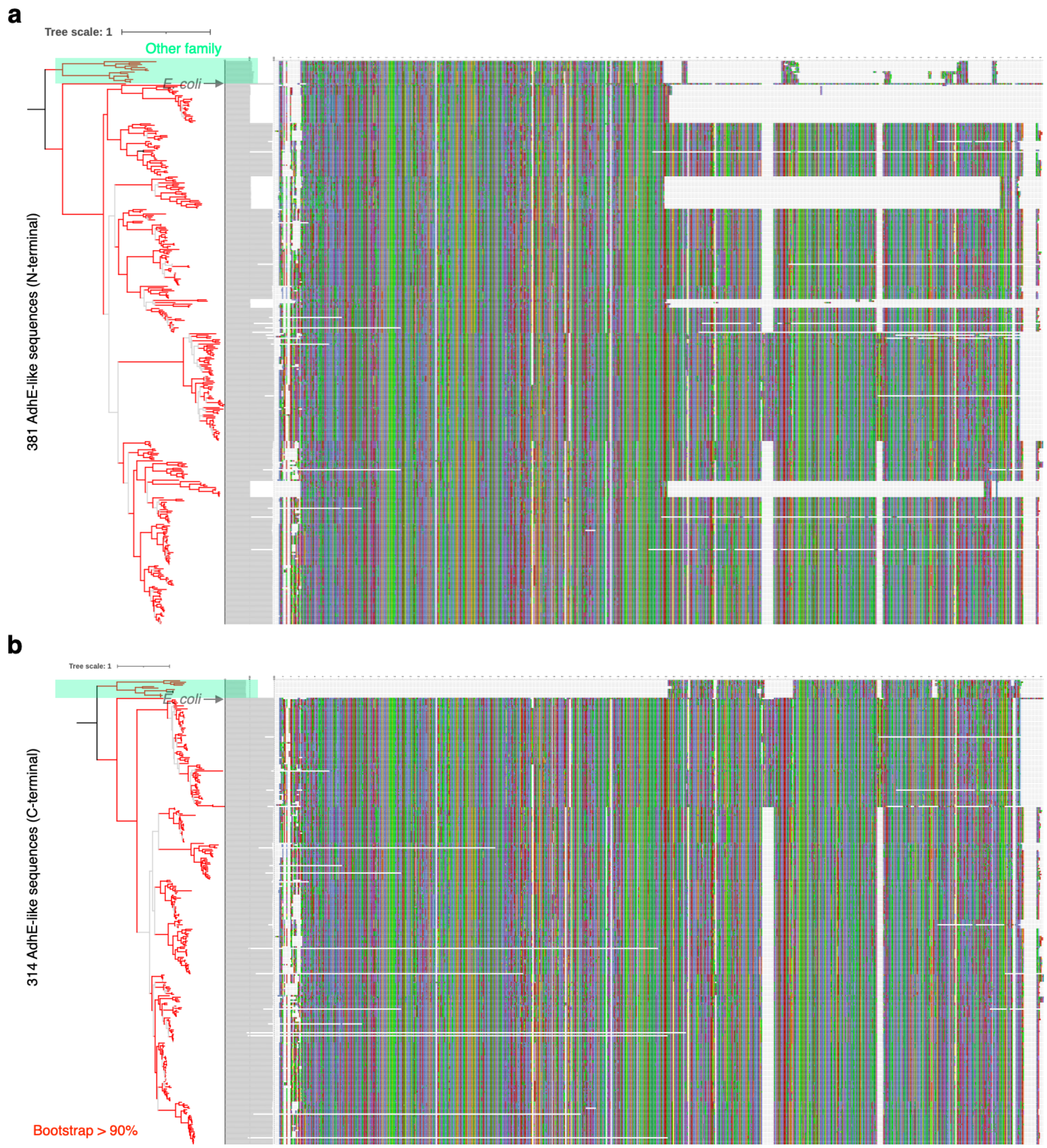

Supplementary Figure 7. Gene phylogenies and the sequences of complete and partial AdhEs. a, **b**, The gene phylogenies and the corresponding multiple sequence alignments of complete and partial AdhEs containing N-terminal half (**a**) or C-terminal half (**b**). The phylogenies were the same trees as **Figure 3c**.

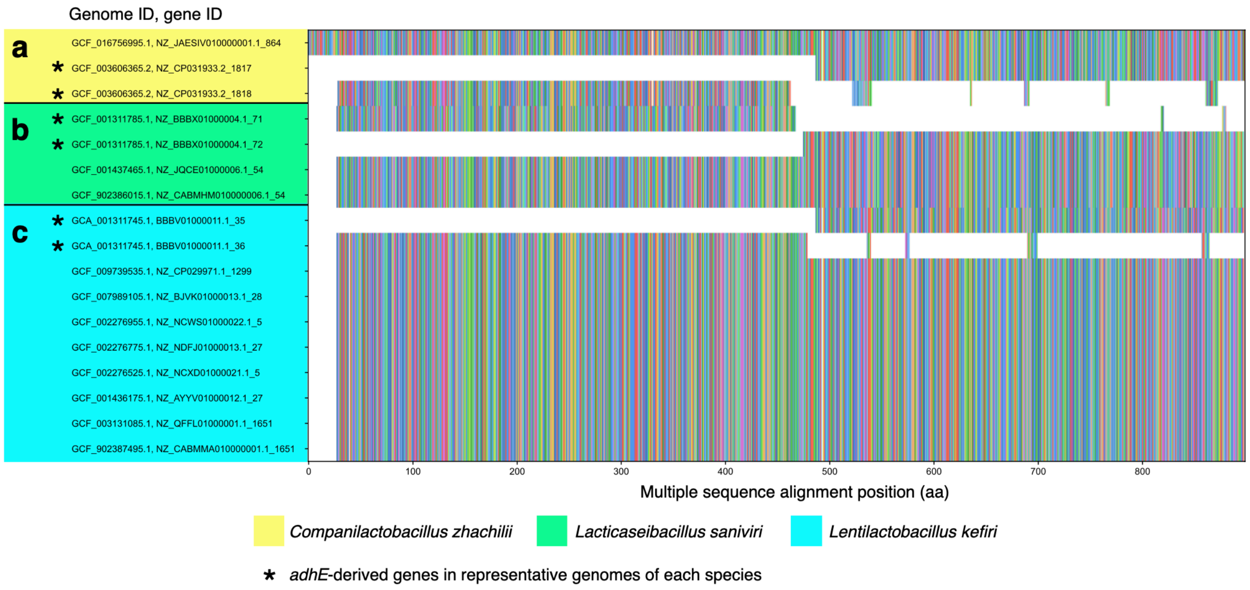

Supplementary Figure 8. Multiple sequence alignment of *adhE* genes in the representative and non-representative genomes of the three species whose representative genomes possessed split *adhE* genes. The alignment of complete and split *adhE* genes detected in representative/non-representative genomes of the three species: *Complanilactobacillus zhachilii* (**a**), *Lacticaseibacillus saniviri* (**b**), and *Lentilactobacillus kefiri* (**c**). Each row represents a gene, and the y-axis label shows the accession number of corresponding genomes and gene IDs. Genes with asterisks are the split *adhE* genes found in representative genomes.

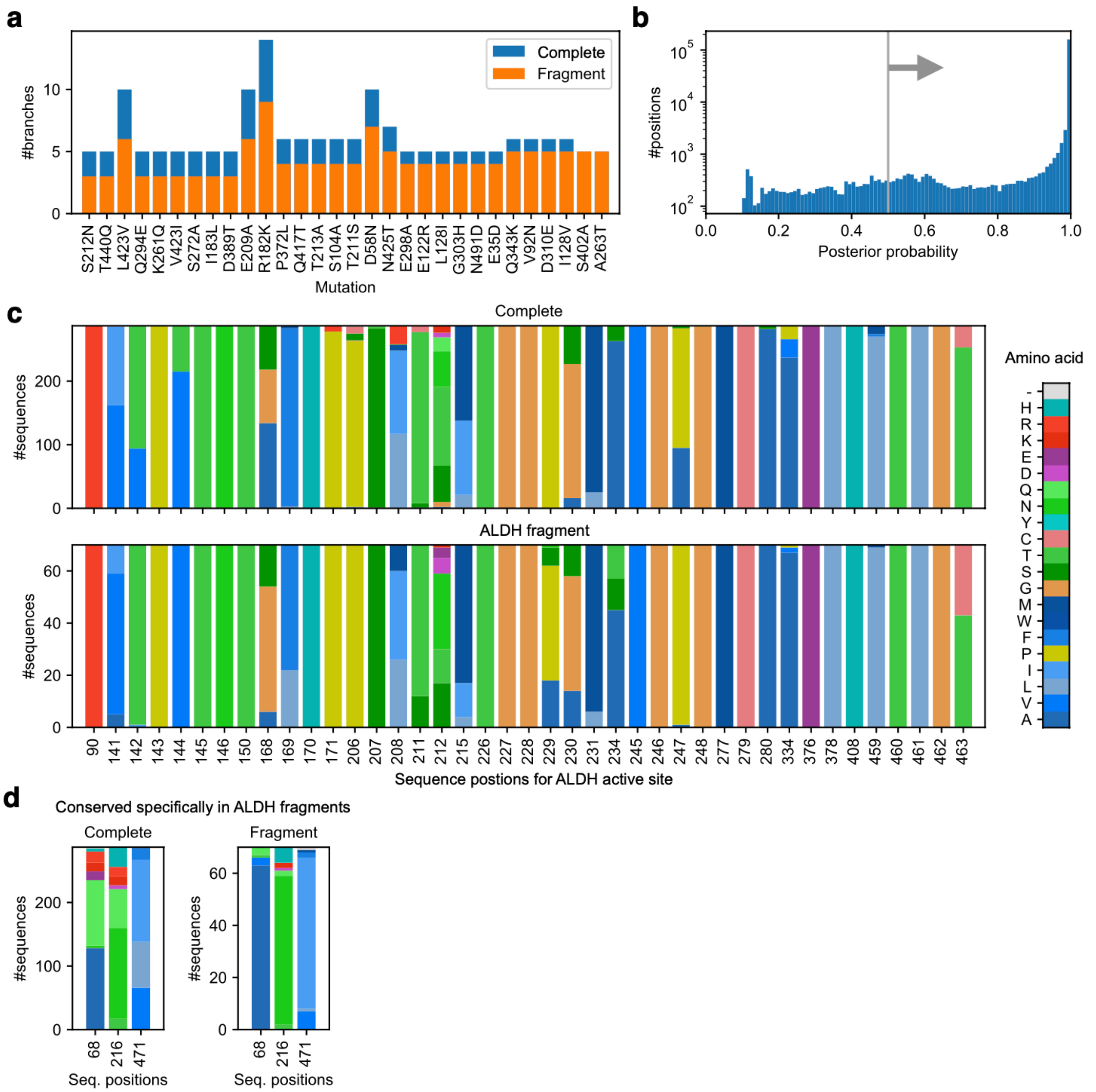

Supplementary Figure 9. Results of ancestral sequence reconstruction and amino acid compositions of complete AdhE and ALDH fragments. a, The distribution of the highest posterior probability estimated for every position of every ancestral sequence. Here we included only sequence positions for which any amino acid (not a gap) was inferred to be the most likely. **b**, Number of branches where every substitution was inferred to happen in complete AdhEs and ALDH fragments. The substitutions (x-axis) were sorted by the ratio of substitution occurrences in ALDH fragments, and only the results for the 15 substitutions with

the highest ratios were shown. **c**, Amino acid composition of the 41 sequence positions for ALDH active site. **d**, Amino acid composition of the three sequence positions where amino acids are divergent in complete AdhEs but common in ALDH fragments.

SUPPLEMENTARY REFERENCES

1. Zheng, J., Ruan, L., Sun, M. & Gänzle, M. A Genomic View of Lactobacilli and Pediococci Demonstrates that Phylogeny Matches Ecology and Physiology. *Appl. Environ. Microbiol.* **81**, 7233–7243 (2015).
