# Supplementary material for: Evolutionary paths toward multi-level convergence of lactic acid bacteria in fructose-rich environments
Source: Commun Biol. 2024 Jul 24;7:902. doi: 10.1038/s42003-024-06580-0 (PMC11269746; doi:10.1038/s42003-024-06580-0)
Supplement: Supplementary file 3 — Description of Additional Supplementary Materials [file 42003_2024_6580_MOESM3_ESM.pdf]

Description of Additional Supplementary Files

File name: Supplementary Data 1

Description: Amino acid sequences of adhE-like genes

File name: Supplementary Data 2

Description: the list of genome accessions for Lactobacillaceae species for which we analyzed their genomes and habitats

File name: Supplementary Data 3

Description: the list of 137 orthologs commonly and independently lost in two FLAB lineages

File name: Supplementary Data 4

Description: The source data behind figures in this paper
